# Prednisolone has the same cardiovascular risk profile as hydrocortisone in glucocorticoid replacement

**DOI:** 10.1530/EC-17-0257

**Published:** 2017-09-29

**Authors:** David J F Smith, Hemanth Prabhudev, Sirazum Choudhury, Karim Meeran

**Affiliations:** 1 Department of EndocrinologyImperial College Healthcare NHS Trust, London, UK; 2 Department of Clinical BiochemistryImperial College Healthcare NHS Trust, London, UK; 3 Department of Investigative MedicineDivision of Diabetes, Endocrinology and Metabolism, Imperial College London, London, UK

**Keywords:** prednisolone, hydrocortisone, adrenal insufficiency, cardiovascular risk

## Abstract

**Introduction:**

Patients who need glucocorticoid replacement in both primary and secondary adrenal insufficiency (AI) have the choice of either once-daily prednisolone or thrice-daily hydrocortisone. A recent European study found no difference between prednisolone and hydrocortisone users in several markers including glucose, weight, body mass index, systolic and diastolic blood pressure and waist circumference, although an increase in cholesterol and low-density lipoprotein (LDL) was suggested in a subgroup of these patients. The aim of this study was to expand the evidence base for the use of these agents as replacement therapy.

**Methods:**

Data from 82 patients on hydrocortisone and 64 patients on prednisolone for AI at Imperial College Healthcare NHS Trust were analysed.

**Results:**

There was no significant difference in total cholesterol, LDL levels or any other risk factors between hydrocortisone and prednisolone patients. Prednisolone was subjectively significantly more convenient than hydrocortisone (*P* = 0.048).

**Conclusions:**

Prednisolone once daily is more convenient than hydrocortisone thrice daily, and there is no difference in the markers of cardiovascular risk measured. Because prednisolone mimics the circadian rhythm better than other glucocorticoids, it should be considered as an alternative to hydrocortisone for AI.

## Introduction

Adrenal insufficiency (AI) is caused either by primary adrenal failure or secondary impairment of the hypothalamic–pituitary–adrenal axis (1). Both result in glucocorticoid deficiency with additional impairment of mineralocorticoid production in primary adrenal failure. The mainstay of treatment is glucocorticoid replacement, with either hydrocortisone or prednisolone (2). Both work by binding to the glucocorticoid receptor (GR) for which prednisolone has the greater avidity (3).

Glucocorticoids in excess have a well-recognised side effect profile, commonly resulting in weight gain, hypertension, early onset diabetes and psychiatric symptoms. These are frequently seen in inflammatory or autoimmune conditions in which treatment with supraphysiological doses of exogenous steroid is required. The aim of glucocorticoid replacement therapy in adrenal failure is to reverse the deficiency using only physiological doses of steroids. Reproducing the diurnal cortisol profile with oral medication is a significant challenge because normal cortisol production is pulsatile and consists of a circadian rhythm and an ultradian rhythm (3, 4, 5). Under-replacement may cause lethargy and an increased risk of Addisonian crises, whereas excessive replacement puts patients at the risk of Cushingoid symptoms and cardiovascular disease (6, 7). In an attempt to mimic circadian rhythmicity, hydrocortisone analogues have been developed, including dual release hydrocortisone (Duocort) (8) and delayed release hydrocortisone (Chronocort) (9). The use of subcutaneous pumps for hydrocortisone delivery has also been attempted with variable success (10). Hydrocortisone is currently the default choice for cortisol replacement as it is identical to the cortisol secreted by the adrenal glands. *In vitro* studies of the GR have further suggested that the synthetic steroids such as dexamethasone and prednisolone alter the normal transcription processes within target cells as a result of their greater avidity for the GR (4). In particular, GRs activated by synthetic glucocorticoids require significantly more time to dissociate from nuclear promoters, suggesting that steroid effects may be seen long after the synthetic glucocorticoid has been washed out.

However, hydrocortisone possesses a short half-life which prevents once-daily oral administration (11). Hydrocortisone must be taken thrice a day to ensure sufficient trough levels, but this comes at the cost of producing post-dose peaks that are not physiological, and cumulatively results in excess steroid exposure. Most patients taking hydrocortisone for AI are either over- or under-treated (11, 12). The risks of over-replacement have not been fully elucidated until recently, and are often overlooked due to an appropriate fear of Addisonian crises. However, evidence of harm from a minor excess of cortisol is apparent from patients who have subclinical autonomous cortisol production with an associated increase in morbidity and mortality from cardiovascular disease (13, 14).

Although prednisolone mimics the physiological cortisol profile more closely than hydrocortisone (15, 16), there is no evidence at present as to which steroid is more appropriate to treat AI. In the absence of such data, we offer patients either hydrocortisone (10 + 5 + 5 mg daily) or prednisolone (2–4 mg once daily). For convenience, some patients now choose the latter. To optimise the dose, patients are offered a hydrocortisone day curve (17) or a prednisolone level. We have set up our own prednisolone assay and aim for an eight-hour trough level of between 15 and 25 µg/L (http://www.imperialendo.com/prednisolone, accessed 28th July 2017) (15).

All patients on steroid replacement are regularly assessed for cardiovascular risk, as this is the commonest cause of premature death in this group (18). A recent European report found that most markers of cardiovascular risk were the same in patients on prednisolone and hydrocortisone, except for total cholesterol (TC) and low-density lipoprotein (LDL), where the values were higher in the prednisolone cohort (19). We have therefore collected data from Imperial College Healthcare NHS Trust, a tertiary centre to compare a more homogeneous group of patients on prednisolone or hydrocortisone replacement.

## Methods

Data were collected from patients who were reviewed between December 2016 and May 2017 taking either prednisolone or hydrocortisone as glucocorticoid replacement therapy for primary or secondary AI at Imperial College Healthcare NHS Trust in London. Patients were between the ages of 18 and 80, had been taking the relevant steroid for more than one month and were not using any other glucocorticoids concurrently. Individuals taking glucocorticoids for suppression of autoimmune disease or other systemic disease were excluded, as were people with congenital adrenal hyperplasia. After applying these criteria, we obtained data from 146 patients, 82 of whom were taking hydrocortisone, with 64 on prednisolone. In order to ensure that there was no age bias, we also carried out a subanalysis of patients aged 18–65. All patients had gone through a normal puberty. Consent was obtained from each patient after full explanation of the purpose and nature of all procedures. As this was a study of normal patient care, and as no intervention was carried out for the purpose of this audit, ethics committee approval was not required.

We used data obtained from our routine clinical screening, analysing parameters including blood pressure, body mass index (BMI), waist-to-hip circumference ratio, lipid profile, glycosylated haemoglobin, random glucose, patient satisfaction, frequency of type 2 diabetes diagnoses and frequency of diagnosed hypertension.

The Shapiro–Wilk test was used to check for the data being normally distributed. Where data were normally distributed, Levene’s test was employed to confirm homogeneity of variances between prednisolone and hydrocortisone groups, prior to subsequent analysis using Student’s *t*-test (alpha level 0.05). The Mann–Whitney *U*-test was used to assess all other non-parametric data (alpha level 0.05). Data were collected and collated into Microsoft Excel 2016 (Microsoft, released 2015). Further statistical analysis was performed using IBM SPSS Statistics for Windows, Version 24.0 (IBM, released 2016).

## Results

**Figure 1:**
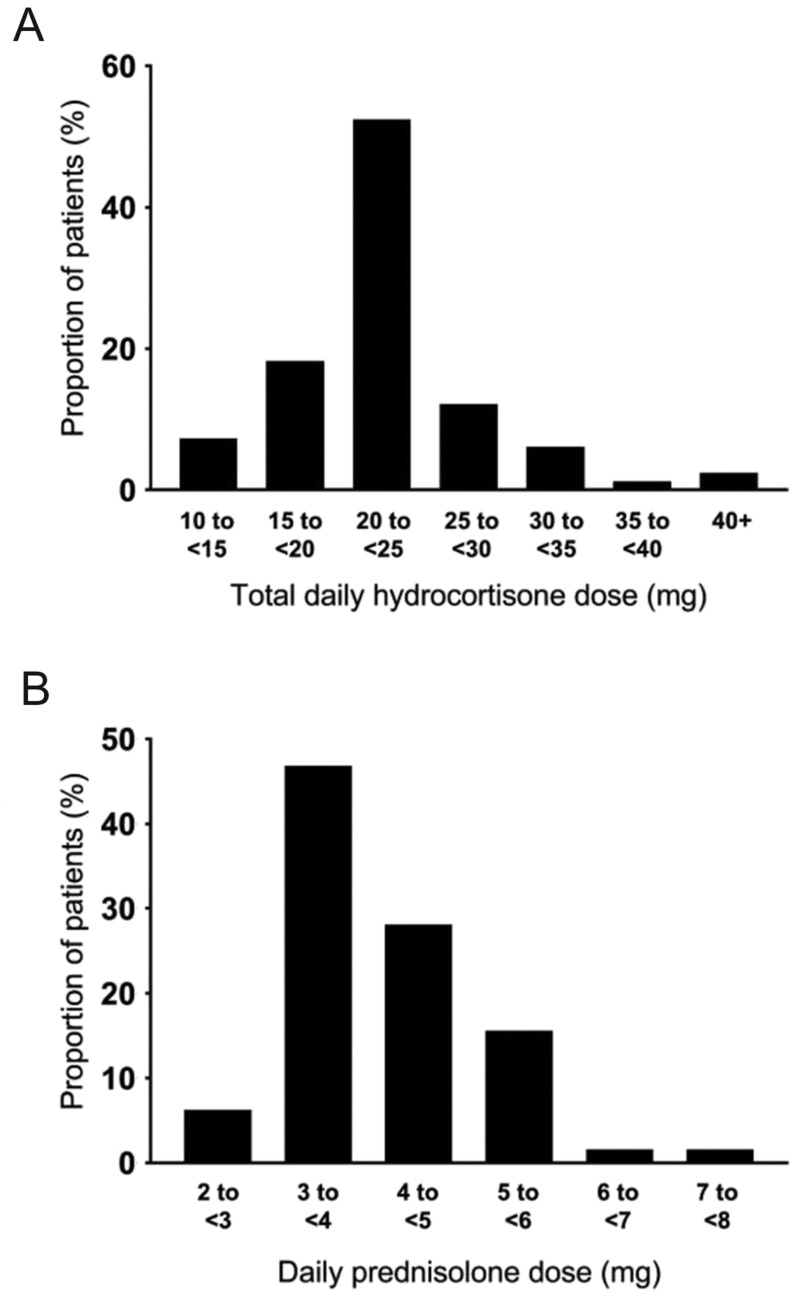
Frequency of the daily doses of replacement therapy taken by patients. (A) Total daily hydrocortisone dose by patients with primary and secondary AIs. (B) Daily dose of prednisolone taken by patients with primary and secondary AIs.

The baseline demographic data are shown in Table 1. The proportion of patients taking anti-hypertensives or statins was similar between the two groups, as were diagnoses of diabetes and hypertension. The proportion of patients with primary AI vs secondary AI was equivalent in both treatment groups. The mean total daily cumulative dose of hydrocortisone was 20.5 mg, while the mean dose of prednisolone was 3.7 mg taken once daily (Table 2). The mean hydrocortisone doses used in cases of primary AI and secondary AI were 22.3 mg and 19.9 mg, respectively. In the prednisolone group, the mean dose in primary AI was 3.9 mg compared to 3.6 mg in secondary AI. There was no difference in hydrocortisone or prednisolone doses between patients with primary and secondary AI. The distribution of doses of each drug is depicted in Fig. 1 (A and B). Two patients who were on hydrocortisone at first review chose to switch to prednisolone, so their data were included in both groups.

**Table 1:** Demographics and characteristics of patients taking glucocorticoids as replacement therapy.

	**Hydrocortisone**	**Prednisolone**
Total patients	82	64
Mean age (s.d.)	57.3 (16.0)	52.2 (15.7)
Median age	58.0 (IQR-23)	53.5 (IQR-26)
Female (%)	62	53
Type 2 diabetes mellitus (%)	22	19
Hypertension (%)	22	22
Anti-hypertensives (%)	34	26
Statins (%)	34	25
Secondary AI (%)	74	83

**Table 2:** Cardiovascular risk factors of patients taking glucocorticoids as replacement therapy.

	**Hydrocortisone** (*n* = 82)	**Prednisolone** (*n* = 64)	***P*-Value**
Total daily dose (mg)	20.5 (*n*** **=** **82)	3.7 (*n*** **=** **64)	
Satisfaction rating	3.7 (1.2) (*n*** **=** **82)	4.1 (0.9) (*n*** **=** **63)	0.048*
Systolic blood pressure (mmHg)	129 (19) (*n*** **=** **82)	127 (18) (*n*** **=** **64)	0.579
Diastolic blood pressure (mmHg)	79 (11) (*n*** **=** **82)	77 (9) (*n*** **=** **64)	0.186
Waist circumference (cm)	101 (18) (*n*** **=** **79)	97 (13) (*n*** **=** **61)	0.354
Hip circumference (cm)	107 (15) (*n*** **=** **80)	105 (11) (*n*** **=** **61)	0.860
Waist-to-hip ratio	0.95 (0.09) (*n*** **=** **79)	0.92 (0.07) (*n*** **=** **61)	0.047*
Weight (kg)	79.8 (16.7) (*n*** **=** **82)	79.6 (15.4) (*n*** **=** **64)	0.884
Height (m)	1.67 (0.09) (*n*** **=** **80)	1.68 (0.12) (*n*** **=** **62)	0.438
Body mass index (kg/m^2^)	28.8 (6.1) (*n*** **=** **80)	28.3 (5.3) (*n*** **=** **62)	0.890
HbA1c (mmol/mol)	42.7 (14.0) (*n*** **=** **78)	41.0 (11.4) (*n*** **=** **62)	0.389
Total cholesterol (mmol/L)	5.15 (1.35) (*n*** **=** **81)	4.77 (1.06) (*n*** **=** **63)	0.067
High density lipoprotein (mmol/L)	1.43 (0.44) (*n*** **=** **81)	1.33 (0.37) (*n*** **=** **63)	0.202
Low-density lipoprotein (mmol/L)	2.90 (1.10) (*n*** **=** **78)	2.75 (0.89) (*n*** **=** **63)	0.450
Random glucose (mmol/L)	6.4 (3.1) (*n*** **=** **82)	5.9 (3.0) (*n*** **=** **63)	0.106

Results are expressed as mean (s.d.). Diastolic blood pressure, height and total cholesterol were assessed using Student’s *t*-test. All other data were compared using the Mann–Whitney *U*-test. Satisfaction ratings (1-very unhappy, 2-not happy, 3-neutral, 4-happy, 5-very happy).

* *P*-Value <0.05.

Our study has found no significant difference in any cardiovascular risk factors between patients taking either prednisolone or hydrocortisone replacement, apart from a slightly lower waist-to-hip ratio (WHR) in patients on prednisolone (Table 2). In particular, there was no difference in LDL or TC. We also noted significantly higher subjective satisfaction scores in the prednisolone cohort (Tables 2 and 3). The subgroup analysis of patients between 18 and 65 also found no difference in any of the factors in Table 2, between patients on prednisolone and hydrocortisone (Table 4).

**Table 3:** Satisfaction ratings (1-very unhappy, 2-not happy, 3-neutral, 4-happy, 5-very happy).

	**Hydrocortisone** (*n* = 82)	**Prednisolone** (*n* = 63)
Happy (score =/>4) (%)	58	73
Unhappy (score =/<2) (%)	16	3

**Table 4:** Subgroup analysis involving participants between 18 and 65.

	Hydrocortisone (*n* = 55)	Prednisolone (*n* = 49)	***P***-Value
Mean age	48.7 (11.7)	45.9 (12.0)	
Median age	52 (IQR-21.5)	47 (IQR-17.0)	
Total daily dose (mg)	21.2 (5.9) (*n*** **=** **55)	3.7 (1.0) (*n*** **=** **49)	
Satisfaction rating	3.7 (1.3) (*n*** **=** **55)	4.0 (0.9) (*n*** **=** **49)	0.200
Systolic blood pressure (mmHg)	124 (17) (*n*** **=** **55)	122 (14) (*n*** **=** **49)	0.483
Diastolic blood pressure (mmHg)	80 (11) (*n*** **=** **55)	76 (9) (*n*** **=** **49)	0.094
Waist circumference (cm)	100 (20) (*n*** **=** **54)	95 (14) (*n*** **=** **47)	0.509
Hip circumference (cm)	108 (17) (*n*** **=** **54)	104 (10) (*n*** **=** **47)	0.638
Waist-to-hip ratio	0.93 (0.10) (*n*** **=** **54)	0.91 (0.08) (*n*** **=** **47)	0.210
Weight (kg)	80.7 (17.6) (*n*** **=** **55)	81.2 (16.3) (*n*** **=** **49)	0.656
Height (m)	1.67 (0.08) (*n*** **=** **54)	1.70 (0.12) (*n*** **=** **48)	0.119
Body mass index (kg/m^2^)	29.2 (6.9) (*n*** **=** **54)	28.1 (5.8) (*n*** **=** **48)	0.608
HbA1c (mmol/mol)	43.5 (16.5) (*n*** **=** **53)	38.3 (8.3) (*n*** **=** **49)	0.211
Total cholesterol (mmol/L)	5.29 (1.36) (*n*** **=** **54)	4.88 (1.11) (*n*** **=** **49)	0.101
High density lipoprotein (mmol/L)	1.47 (0.45) (*n*** **=** **54)	1.33 (0.38) (*n*** **=** **49)	0.127
Low-density lipoprotein (mmol/L)	3.05 (1.14) (*n*** **=** **52)	2.90 (0.88) (*n*** **=** **49)	0.453
Random glucose (mmol/L)	6.3 (3.3) (*n*** **=** **55)	5.5 (2.8) (*n*** **=** **49)	0.104

Results are expressed as mean (s.d.) unless otherwise stated. Systolic blood pressure, diastolic blood pressure, height and total cholesterol were assessed using Student’s *t*-test. All other data were compared using the Mann–Whitney *U*-test. Satisfaction ratings (1-very unhappy, 2-not happy, 3-neutral, 4-happy, 5-very happy).

## Discussion

This retrospective observational study suggests that hydrocortisone and prednisolone are equivalent steroid replacement therapies with no evidence that one drug possesses a bigger cardiovascular risk than the other. While the satisfaction scores were higher in the prednisolone cohort, this finding should be viewed with caution due to the potential for variability in questioning and the fact that it may be influenced by the convenience of once-daily prednisolone dosing rather than superior control of symptoms. Similarly, the better WHR in patients on prednisolone is unlikely to be clinically significant given the lack of difference in the other parameters. It is possible that patients on hydrocortisone have been on this replacement longer than those on prednisolone. As we have only been using prednisolone at our centre since 2014, long-term effects may be yet to develop.

The comparison of the two drugs in glucocorticoid replacement is a relatively unexplored area, although a cross-sectional study in 2008 showed no difference in subjective health status between 409 patients taking either prednisolone or hydrocortisone replacement (20). A more recent study also found no significant differences in the common side effects of glucocorticoids (blood pressure, HbA1c, BMI, WHR) (19), although in a subgroup of patients, higher LDL and TC levels were found in individuals taking prednisolone. It was concluded that individuals taking prednisolone therefore had a higher relative cardiovascular risk. Most patients were, however, receiving an excess of prednisolone (5–6 mg), and the data for this parameter were incomplete, being derived from only 31 patients. Furthermore, the data were collected from different centres across Europe creating exposure to confounding factors such as patient groups in one country on one drug being compared with a group elsewhere on a different drug. This was seen in a cross-sectional study comparing the two steroids, where patients in West Germany were largely treated with hydrocortisone, while those in East Germany were taking prednisolone (20). Each group will be subject to their own genotypic, phenotypic and socioeconomic influences, and consequently the results may be confounded by external factors such as genetics, wealth and standards of healthcare. We were able to minimise such variance by examining a homogeneous population who attend the pituitary or adrenal services at Imperial College Healthcare NHS Trust in London. We were also able to obtain a more complete set of data with a similar number of patients on each drug, allowing for a more objective comparison, and had values for the majority of patients in all parameters which we sought to measure. Using the same parameters as Quinkler and coworkers (19) in a more homogenous population, we have not observed the same significant differences in TC or LDL and consequent relative cardiovascular risk.

An important issue raised by our study and the one conducted by Quinkler and coworkers (19) is that of glucocorticoid dosing. The discovery of glucocorticoids in the 1940s converted conditions such as Addison’s disease and many autoimmune diseases into conditions that were no longer rapidly fatal (21). Doctors have tended to over-prescribe steroids to prevent Addisonian crises without recognising the side effects of the excess (6). However, we now see an increased risk of cardiovascular death in hypoadrenal patients, probably due to excess cortisol administration, and consequently there has been a fall in the average dose of hydrocortisone prescribed. The guidelines from the endocrine society are to prescribe 3–5 mg of prednisolone daily (2), but it is likely that prednisolone is still prescribed to excess (22) as it has been found to have a potency between six and eight times higher than hydrocortisone (23). At our centre, we have been using low-dose prednisolone as our standard glucocorticoid replacement since 2014. Dosing regimens are guided using serum 8-hour prednisolone trough levels, which has resulted in our finding that low-dose replacement of 2–4 mg once daily is appropriate for most patients, and that 5 mg is excessive (http://www.imperialendo.com/prednisolone, accessed 28th July 2017) (15). The doses of glucocorticoids in this study are more equivalent to physiological requirements than those used by Quinkler and coworkers whose patients were mostly taking 5 mg daily (19).

**Figure 2:**
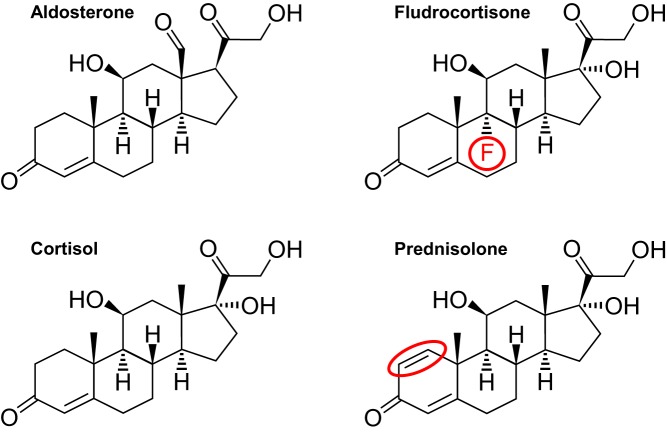
Biochemical structure of aldosterone, fludrocortisone, cortisol and prednisolone. The changes that give a longer half-life are shown in red. A fluorine atom is present in fludrocortisone, and a double bond in prednisolone is the only difference between these molecules and cortisol.

Hydrocortisone is the native hormone cortisol, whereas prednisolone is an analogue with a double bond between positions 1 and 2 (24, 25) (Fig. 2). The use of analogues in replacement therapy is well established in modern medicine, with fludrocortisone and insulin analogues (such as insulin glargine) commonly used due to their longer half-life in comparison with native hormones. Using prednisolone in replacement therapy should have the same benefit, as the increased binding and slower dissociation (4) may reduce the risk of Addisonian crises. Hydrocortisone usage can be associated with peak levels above physiological requirements and troughs below them (11).

In view of the longer duration of action and the ease of administration, we have been using low-dose prednisolone as our standard glucocorticoid replacement. Our findings of a similarity in side effect profiles reaffirm our preference, although these results should be interpreted with caution in view of the fact that this is a retrospective study. Blood pressure was measured at a single time point in the outpatient clinic, potentially missing the nocturnal blood pressure dip as assessed by ambulatory blood pressure monitoring. Furthermore, data were not collected to exclude participants with a familial tendency for dyslipidaemia or dysglycaemia, and analysis was not corrected for lipid lowering medication or anti-diabetic medication. A double-blind randomised controlled trial is required in order to determine whether there is any statistically significant difference in the prevalence of adverse effects of the two glucocorticoids. In the absence of evidence demonstrating superiority of one treatment above another, it is the opinion of the authors that individuals with AI should be commenced on prednisolone 3–4 mg daily, and the dose adjusted with 8-h prednisolone levels and day curves (15).

## Declaration of interest

The authors declare that there is no conflict of interest that could be perceived as prejudicing the impartiality of the research reported.

## Funding

This work did not receive any specific grant from any funding agency in the public, commercial or not-for-profit sector.

## References

[1] Arlt W Allolio B Bidlingmaier F Klingmuller D Bengtsson B Ohnhaus E. Adrenal insufficiency. Lancet 2003 361 1881–1893. (10.1016/S0140-6736(03)13492-7) 12788587

[2] Bornstein SR Allolio B Arlt W Barthel A Don-Wauchope A Hammer GD Husebye ES Merke DP Murad MH Stratakis CA Torpy DJ. Diagnosis and treatment of primary adrenal insufficiency: an endocrine society clinical practice guideline. Journal of Clinical Endocrinology and Metabolism 2016 101 364–389. (10.1210/jc.2015-1710) 26760044 PMC4880116

[3] Lan NC Graham B Bartter FC Baxter JD. Binding of steroids to mineralocorticoid receptors: implications for in vivo occupancy by glucocorticoids. Journal of Clinical Endocrinology and Metabolism 1982 54 332–342. (10.1210/jcem-54-2-332) 6274900

[4] Stavreva DA Wiench M John S Conway-Campbell BL McKenna MA Pooley JR Johnson TA Voss TC Lightman SL Hager GL. Ultradian hormone stimulation induces glucocorticoid receptor-mediated pulses of gene transcription. Nature Cell Biology 2009 11 1093–1102 (10.1038/ncb1922) 19684579 PMC6711162

[5] Chrousos GP. Editorial: ultradian, circadian, and stress-related hypothalamic-pituitary-adrenal axis activity – a dynamic digital-to-analog modulation. Endocrinology 1998 139 437–440. (10.1210/endo.139.2.5857) 9449607

[6] Behan LA Carmody D Rogers B Hannon MJ Davenport C Tormey W Smith D Thompson CJ Stanton A Agha A. Low-dose hydrocortisone replacement is associated with improved arterial stiffness index and blood pressure dynamics in severely adrenocorticotrophin-deficient hypopituitary male patients. European Journal of Endocrinology 2016 174 791–799. (10.1530/EJE-15-1187) 27025241

[7] Sherlock M Reulen RC Alonso AA Ayuk J Clayton RN Sheppard MC Hawkins MM Bates AS Stewart PM. ACTH deficiency, higher doses of hydrocortisone replacement, and radiotherapy are independent predictors of mortality in patients with acromegaly. Journal of Clinical Endocrinology and Metabolism 2009 94 4216–4223. (10.1210/jc.2009-1097) 19808848

[8] Johannsson G Nilsson AG Bergthorsdottir R Burman P Dahlqvist P Ekman B Engstrom BE Olsson T Ragnarsson O Ryberg M Improved cortisol exposure-time profile and outcome in patients with adrenal insufficiency: a prospective randomized trial of a novel hydrocortisone dual-release formulation. Journal of Clinical Endocrinology and Metabolism 2012 97 473–481. (10.1210/jc.2011-1926) 22112807

[9] Whitaker M Debono M Huatan H Merke D Arlt W Ross RJ. An oral multiparticulate, modified-release, hydrocortisone replacement therapy that provides physiological cortisol exposure. Clinical Endocrinology 2014 80 554–561. (10.1111/cen.12316) 23980724 PMC3937303

[10] Oksnes M Bjornsdottir S Isaksson M Methlie P Carlsen S Nilsen RM Broman JE Triebner K Kampe O Hulting AL Continuous subcutaneous hydrocortisone infusion versus oral hydrocortisone replacement for treatment of Addison’s disease: a randomized clinical trial. Journal of Clinical Endocrinology and Metabolism 2014 99 1665–1674. (10.1210/jc.2013-4253) 24517155

[11] Simon N Castinetti F Ouliac F Lesavre N Brue T Oliver C. Pharmacokinetic evidence for suboptimal treatment of adrenal insufficiency with currently available hydrocortisone tablets. Clinical Pharmacokinetics 2010 49 455–463. (10.2165/11531290-000000000-00000) 20528006

[12] Debono M Ross RJ. What is the best approach to tailoring hydrocortisone dose to meet patient needs in 2012? Clinical Endocrinology 2013 78 659–664. (10.1111/cen.12117) 23194144

[13] Debono M Bradburn M Bull M Harrison B Ross RJ Newell-Price J. Cortisol as a marker for increased mortality in patients with incidental adrenocortical adenomas. Journal of Clinical Endocrinology and Metabolism 2014 99 4462–4470. (10.1210/jc.2014-3007) 25238207 PMC4255126

[14] Tauchmanova L Rossi R Biondi B Pulcrano M Nuzzo V Palmieri EA Fazio S Lombardi G. Patients with subclinical Cushing’s syndrome due to adrenal adenoma have increased cardiovascular risk. Journal of Clinical Endocrinology and Metabolism 2002 87 4872–4878. (10.1210/jc.2001-011766) 12414841

[15] Williams EL Choudhury S Tan T Meeran K. Prednisolone replacement therapy mimics the circadian rhythm more closely than other glucocorticoids. Journal of Applied Laboratory Medicine 2016 1 152–161. (10.1373/jalm.2016.020206) 33626793

[16] Huseman CA Varma MM Blizzard RM Johanson A. Treatment of congenital virilizing adrenal hyperplasia patients with single and multiple daily doses of prednisone. Journal of Pediatrics 1977 90 538–542. (10.1016/S0022-3476(77)80362-4) 839364

[17] Howlett TA. An assessment of optimal hydrocortisone replacement therapy. Clinical Endocrinology 1997 46 263–268. (10.1046/j.1365-2265.1997.1340955.x) 9156032

[18] Bergthorsdottir R Leonsson-Zachrisson M Oden A Johannsson G. Premature mortality in patients with Addison’s disease: a population-based study. Journal of Clinical Endocrinology and Metabolism 2006 91 4849–4853. (10.1210/jc.2006-0076) 16968806

[19] Quinkler M Ekman B Marelli C Uddin S Zelissen P Murray RD. Prednisolone is associated with a worse lipid profile than hydrocortisone in patients with adrenal insufficiency. Endocrine Connections 2017 6 1–8. (10.1530/EC-16-0081) 27864317 PMC5148794

[20] Bleicken B Hahner S Loeffler M Ventz M Allolio B Quinkler M. Impaired subjective health status in chronic adrenal insufficiency: impact of different glucocorticoid replacement regimens. European Journal of Endocrinology 2008 159 811–817. (10.1530/EJE-08-0578) 18819943

[21] Erichsen MM Løvås K Fougner KJ Svartberg J Hauge ER Bollerslev J Berg JP Mella B Husebye ES. Normal overall mortality rate in Addison’s disease, but young patients are at risk of premature death. European Journal of Endocrinology 2009 160 233–237. (10.1530/EJE-08-0550) 19011006

[22] Quinkler M Ekman B Marelli C Uddin S Zelissen P Murray R. Hormone replacement therapy with prednisolone in adrenal insufficiency patients: data from the European Adrenal Insufficiency Registry (EuAIR). Endocrine Abstracts 2015 38 P410 (10.1530/endoabs.38.P410)

[23] Caldato MC Fernandes VT Kater CE. One-year clinical evaluation of single morning dose prednisolone therapy for 21-hydroxylase deficiency. Arquivos Brasileiros de Endocrinologia e Metabologia 2004 48 705–712. 15761542 10.1590/s0004-27302004000500017

[24] Boland EW. 6a-Methyl corticosteroids a new series of anti-inflammatory compounds; clinical appraisal of their antirheumatic potencies. California Medicine 1958 88 417–422. 13536856 PMC1512303

[25] Pechet MM Bowers B Bartter FC. Metabolic studies with a new series of 1, 4-diene steroids. i. effects in Addisonian subjects of prednisone, prednisolone and the 1,2-dehydro analogues of corticosterone, desoxycorticosterone, 17-hydroxy-11-desoxycorticosterone, and 9 alpha-fluorocortisol. Journal of Clinical Investigation 1959 38 681–690. (10.1172/JCI103847) 13641420 PMC293208

